# Mesenchymal stem cell-derived extracellular vesicles: a regulator and carrier for targeting bone-related diseases

**DOI:** 10.1038/s41420-024-01973-w

**Published:** 2024-05-02

**Authors:** Jiandong Tang, Xiangyu Wang, Xu Lin, Chao Wu

**Affiliations:** Orthopaedics Center, Zigong Fourth People’s Hospital, Tan mu lin Street 19#, Zigong, 643099 Sichuan Province China

**Keywords:** Non-coding RNAs, Stem-cell differentiation, Drug delivery

## Abstract

The escalating threat of bone-related diseases poses a significant challenge to human health. Mesenchymal stem cell (MSC)-derived extracellular vesicles (MSC-EVs), as inherent cell-secreted natural products, have emerged as promising treatments for bone-related diseases. Leveraging outstanding features such as high biocompatibility, low immunogenicity, superior biological barrier penetration, and extended circulating half-life, MSC-EVs serve as potent carriers for microRNAs (miRNAs), long no-code RNAs (lncRNAs), and other biomolecules. These cargo molecules play pivotal roles in orchestrating bone metabolism and vascularity through diverse mechanisms, thereby contributing to the amelioration of bone diseases. Additionally, engineering modifications enhance the bone-targeting ability of MSC-EVs, mitigating systemic side effects and bolstering their clinical translational potential. This review comprehensively explores the mechanisms through which MSC-EVs regulate bone-related disease progression. It delves into the therapeutic potential of MSC-EVs as adept drug carriers, augmented by engineered modification strategies tailored for osteoarthritis (OA), rheumatoid arthritis (RA), osteoporosis, and osteosarcoma. In conclusion, the exceptional promise exhibited by MSC-EVs positions them as an excellent solution with considerable translational applications in clinical orthopedics.

## Facts

- MSC-EVs-loaded non-coding RNAs can regulate bone disease progression.
- MSC-EVs can be used as drug carriers for bone diseases.
- Targeting of MSC-EVs to bone tissue can be increased by engineered modifications.

## Open questions

- How to increase the yield and purity of EVs secreted by MSC?
- Which engineering modification techniques can further improve bone targeting in MSC-EVs?
- How the drug loading efficiency of MSC-EVs can be further improved?

## Introduction

Bones, integral in maintaining body posture, serve not only to protect internal organs and support hematopoiesis but also actively regulate the body’s mineral balance, calcium and phosphorus metabolism, and maintain normal physiological functions [1, 2]. However, the aging process disrupts this delicate balance, leading to the dysregulation of bone metabolism and the development of bone diseases such as osteoporosis, OA, and bone tumors [3]. Conditions like osteosarcoma, malignant tumors with bone metastases, and other imbalances in bone metabolism can result in severe pain and a poor prognosis for patients [4]. Furthermore, orthopedic diseases, including OA, bone injuries, and degenerative disc disease, pose threats to health and reduce the quality of life for millions globally [5]. Current clinical treatments for orthopedic conditions include bed rest, non-steroidal anti-inflammatory drugs (NSAIDs), analgesics, lumbar discectomy, and interbody fusion [6], provide only temporary relief of symptoms and lack precision in targeting the pathogenesis of bone disease [7]. Hence, there is an urgent need to explore the mechanisms of bone diseases and develop safe and effective biomaterials/agents to improve the prognosis of patients with orthopedic diseases.

MSC are known for their pluripotent nature and the ability for self-renewal and multi-lineage differentiation [8], have been utilized in the past, either alone or in combination with biomaterials and growth factors, for treating musculoskeletal disorders [9]. However, the potential risks of immune rejection, tumorigenicity, and induction of tumor resistance associated with MSC transplantation have limited their use in orthopedic disease treatment [10]. Recent studies have revealed that the positive effects of MSC on bone disease may be mediated by paracrine mechanisms, particularly MSC-EVs [11, 12]. EVs represent heterogeneous membrane-bound vesicles of lipid bilayer, released into the microenvironment by all cells [13]. The researchers classified EVs into exosomes (30–150 nm), microvesicles (200–1000 nm) and apoptotic vesicles (800–5000 nm) based on the diameter of the EVs [14], the absence of a uniform nomenclature arises from overlapping characteristics in size, density, contents, and surface molecules among vesicles subtypes [15]. Consequently, for this study, the collective term “EVs” encompasses exosomes, microvesicles, and apoptotic vesicles. EVs serve as carriers for diverse cargo, including protein molecules, nucleic acids, pro-inflammatory factors, cytokines, and transcription factor receptors, enabling their participation in intercellular signaling through receptor-ligand interactions [16]. Furthermore, the genetic material within EVs, such as miRNAs, non-coding RNAs, and DNA, contributes to their involvement in immune response, signal transduction, and antigen presentation and influences cellular physiology and pathology [17, 18]. Functionally mirroring their parental cells, EVs play a crucial role in influencing disease progression by mediating angiogenesis through cell-to-cell communication and modulating immune responses [19].

Mounting evidence suggests the involvement of MSC-EVs in the regulation of bone-related diseases, including OA, fracture healing, degenerative bone diseases, and bone tumors, mediated by paracrine mediators [9, 20, 21]. Leveraging their commendable biocompatibility, tissue penetration, and pro-regenerative abilities akin to parental cells, MSC-EVs are increasingly harnessed as nano drug delivery carriers, finding applications in the treatment of diverse diseases such as oncology, neurodegenerative disorders, and immune disorders [22, 23]. Furthermore, the synergy of MSC-EVs with engineering techniques enhances the precision targeting of drugs for specific diseases [24]. This review provides a comprehensive summary of the regulatory role of MSC-EVs in the progression of orthopedic diseases. It explores their promising clinical application as nanocarriers for the treatment of orthopedic diseases, with the optimistic anticipation that cell-free therapies will pave the way for innovative perspectives in orthopedic disease treatment.

## Regulation of osteoblast biological behavior by MSC-EVs

**Fig. 1:**
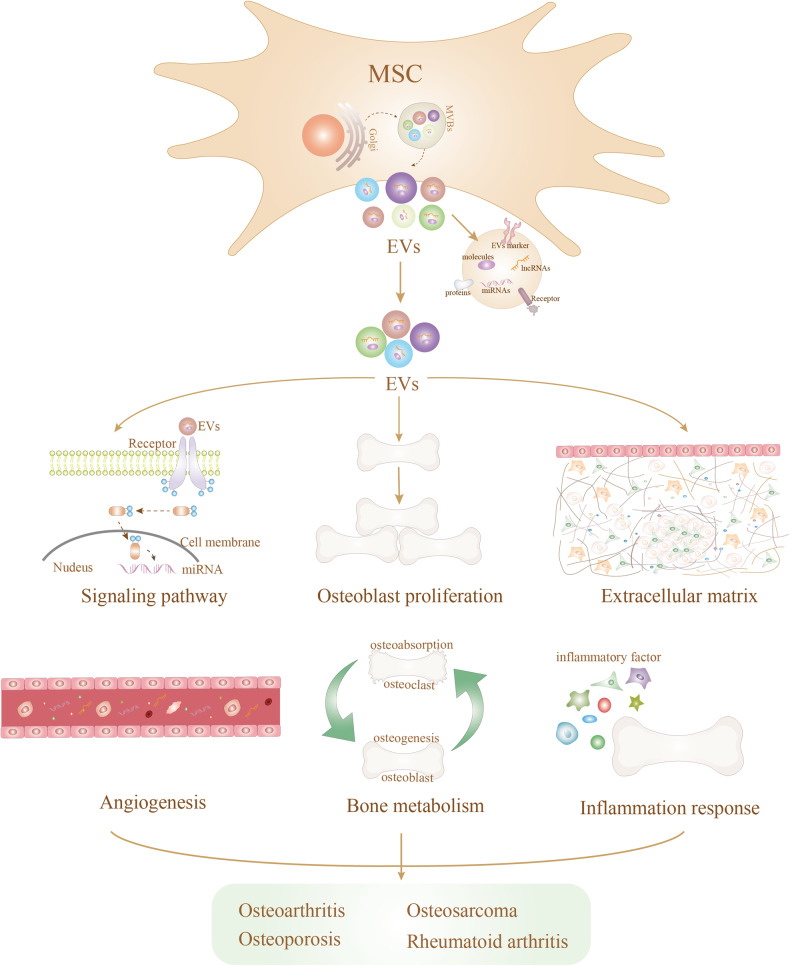
MSC-EVs regulate bone-related disease progression through various mechanisms. These include the regulation of signaling pathways, promotion of osteoblast proliferation, modulation of the extracellular matrix, facilitation of angiogenesis, influence on bone metabolism, and modulation of inflammatory responses.

Operating at the nanoscale, these EVs are ubiquitously secreted by nearly all cell’s physiological or pathological conditions, utilizing endocytosis and biosynthetic pathways [25]. Activation of the endocytosis pathway, triggered by external or internal signals from the local environment, initiates membrane invagination, forming early endosomes [26, 27]. Guided by various cellular signaling pathways, these early endosomes transform into late endosomes. The subsequent fusion of multicellular bodies derived from late endosomes with the cell membrane culminates in the secretion of EVs [25, 28]. EVs, acting as messengers, transport nucleic acids, proteins, and lipids in a paracrine or endocrine manner, thereby regulating the biological functions of recipient cells [29]. Within the bone metabolism microenvironment, MSC-EVs emerge as pivotal players in the regulation of bone-related diseases, because MSC-EVs affect the progression of bone diseases by regulating signaling pathways, bone metabolism, inflammatory responses, angiogenesis, promoting osteoblast proliferation, and inhibiting ECM degradation (Fig. 1).

### Regulating signaling pathway

MSC release EVs that exert a significant influence on the biological behavior of bone cells, thereby impacting the progression of bone diseases. MSC-EVs, enriched with various substances, including miRNAs and proteins, predominantly govern their regulatory effects on osteoblasts through the genetic material that they carry [30, 31]. Notably, specific miRNAs such as miRNA-935, miRNA-361-5p, miR-126, and lncRNA-H19 have demonstrated the ability to mitigate osteoporosis, OA, and bone damage by fostering osteoblast proliferation [32–35]. These signaling molecules (e.g., miRNAs), when introduced into recipient cells, profoundly affect bone formation and the advancement of skeletal diseases. Importantly, variations in genetic information lead to differential expression levels of signaling molecules, such as miRNAs, in osteoblasts [36]. Differences in the expression of miRNAs are evident in MSC-EVs promoting osteogenic differentiation. For instance, miR-135b, miR-203, miR-219, miR-299-5p, and miR-302b are significantly upregulated, while miR-155, miR-885-5p, miR-181a, and miR-320c are downregulated during osteogenic differentiation [37]. Moreover, the same miRNA may exhibit varying expression levels in different bone diseases. Therefore, when investigating bone diseases, it is crucial to consider the different effects of altered expression of signaling molecules from various aspects.

Cell growth regulation involves multiple growth factor receptors, where intracellular kinases activate or phosphorylate these receptors’ structural domains. This activation triggers downstream pro-growth signals through pathways like AKT (protein kinase B), protein kinase C (PKC), and MAP kinase, influencing osteoblast proliferation and disease progression [38, 39]. For instance, MSC-EVs have been shown to alleviate osteoporosis by promoting osteoblast proliferation through the mitogen-activated protein kinase (MAPK) pathway [38]. Additionally, MSC-EVs contribute to spinal cord injury alleviation by inhibiting TLR4/MyD88/NF-κB signaling pathways [40]. The presence of lncRNA XIST in MSC-EVs promotes osteosarcoma growth and metastasis through the miR-655/ACLY signaling pathway [41]. These findings underscore the pivotal role of MSC-EVs in regulating bone diseases [42], and future studies investigating the effects of remaining miRNAs/lncRNAs and other signaling pathways on bone diseases hold promise for advancing the utilization of MSC-EVs in clinical application.

### Promoting osteoblast proliferation

As previously mentioned, MSC-EVs harbor a diverse array of signaling molecules, including proteins, lipids, and miRNAs, capable of influencing osteoblast activity and impacting bone formation. Notably, other signaling molecules may also contribute to the regulation of osteoblast proliferation and differentiation. Li et al. [43] observed that hypoxia inducible factor (HIF)-1α in MSC-EVs stimulated the expression of relevant osteogenic genes, rescuing bone ischemic necrosis by mitigating early steroid induction. Additionally, tsRNA-10277-containing MSC-EVs were found to regulate the lipogenic and osteogenic potential of bone marrow MSC (BM-MSC) [44].

Numerous cell biology studies have elucidated the mechanisms through which BM-MSC or MSC-EVs regulate osteoblast proliferation and differentiation, encompassing the following aspects: (1) EVs can induce the differentiation of BM-MSC into osteoblasts by regulating growth factors, osteogenic proteins, and transcription growth factor-β1 (TGF-β1) [45]. Furthermore, EVs can regulate protein expression through miRNA to facilitate the differentiation of BM-MSC into osteoblasts [46]. (2) MSC-EVs can directly enhance the proliferative capacity of osteoblasts through miRNAs or signaling pathways, thereby alleviating bone disease progression. For example, miR-935 carried by MSC-EVs supports the proliferation and differentiation of osteoblasts by targeting STAT1 [35]. Moreover, miR-1260b derived from MSC-EVs inhibits osteoclast activity, regulating the dynamic balance of osteoblasts through the Wnt5a-mediated RANKL pathway [47]. (3) Osteoblast proliferation and bone formation may be associated with enhanced vascularization, as miR-29a enriched in EVs secreted by BM-MSC promotes angiogenesis by being taken up by human umbilical vein endothelial cells [48]. (4) As mediators of cellular communication, MSC-EVs regulate immune responses and anti-apoptosis through cellular interactions, ultimately favoring osteoblast proliferation and bone formation [49]. For instance, MSC-EVs can alter macrophage polarization phenotypes by activating NF-κB signaling, promoting osteoblast differentiation [50]. Additionally, osteoblast-derived EVs inhibit osteoclast differentiation via the miR-503-3p/Hpse axis, thereby promoting bone formation [51].

The proliferation and differentiation of osteoblasts are thus recognized as dynamic processes involving multiple biological behaviors, where angiogenesis, immune cell phenotype, and bone metabolism play crucial roles.

### Regulating angiogenesis

Angiogenesis plays a critical role in the evolution of musculoskeletal structures and the maintenance of normal physiological function. This process accelerates the delivery of nutrients, oxygen, and cells, thereby upholding the structural and functional integrity of joints and soft tissues. Moreover, angiogenesis facilitates the differentiation and mineralization of cartilage, promoting the establishment of bone homeostasis and bone healing [52]. Recent research suggests that MSC play a role in the in vivo generation of new blood vessels, attributed to their ability to secrete angiogenic factors and proteases, thereby promoting angiogenesis in disease-related signaling [53]. Critical players in this process include matrix metalloproteinase 2 (MMP-2), transforming growth factor-β (TGF-β), interleukin-6 (IL-6), and vascular endothelial growth factor (VEGF) are crucial [54, 55].

Interestingly, mounting evidence suggests that MSC-EVs contribute to the modulation of bone diseases through the remodeling of angiogenesis. Similar to their parental cells, MSC-EVs regulate bone formation via angiogenesis, involving multiple soluble mediators [56]. For instance, MSC-EVs promote angiogenesis through the VEGF and Hippo signaling pathways, enhancing tendon-bone healing [57]. Additionally, lncRNA-H19 in MSC-EVs supports bone formation by activating the ngpt1/Tie2-NO signaling pathway to promote angiogenesis [32]. Notably, VEGF emerges as a key mediator of angiogenesis, with its activity potentially linked to YAP/TAZ signaling and Hippo pathway activation [58]. However, the detailed mechanisms through which MSC-EVs ameliorate bone pathogenesis by promoting angiogenesis warrant further investigation. It would be interesting to explore whether MSC-EVs mediate their effects through specific cytokines (e.g., TGF-β, IL-6). Studies have shown that MSC-EVs promote angiogenesis through MMP-2, and MSC-EVs-derived TGF-β attenuates OA by regulating vascular activity [56, 59]. These findings confirm the potential clinical application of MSC-EVs in alleviating osteoarthropathy by promoting angiogenesis.

### Inhibiting ECM degradation

Collagen, an essential component of tendon and bone, plays an indispensable role in maintaining the normal physiological function of the bone and joint [60]. Inflammatory cytokines, especially interleukin-1β (IL-1β) and tumor necrosis factor-α (TNF-α), stimulate the production of MMPs, leading to the degradation of all components of the extracellular matrix (ECM). This process results in permanent damage to cartilage, tendons, and bones, contributing to the development of diseases such as OA and RA [61]. Previous studies have established that collagenases, especially MMP-1 and MMP-13, play a pivotal role in inducing OA and RA by accelerating collagen degradation [62]. Interestingly, researchers have identified a unique ability of MSC-EVs to inhibit matrix degradation during arthritis progression. MSC-EVs induce the expression of type II collagen and aggregated glycans, expediting cartilage remodeling while simultaneously limiting matrix and collagen catabolism by inhibiting MMP-13 expression [63].

Furthermore, MSC-EVs may suppress osteoarticular inflammation by downregulating the expression of IL-1β, MMP-1, and MMP-13 [64]. Given that the regulation of ECM degradation by MSC-EVs is a multifactorial and correlated process, the activation of specific signaling pathways is equally noteworthy. By inhibiting ECM degradation along with the production of inflammatory factors, MSC-EVs present the possibility of inflammatory factors counteracting their matrix degradation inhibition by promoting ECM degradation [65]. For instance, MSC-EVs inhibit matrix degradation and the production of inflammatory factors through the miRNA-130b-3p-mediated LRP12/AKT/β-catenin axis, thereby alleviating OA progression [65]. Similarly, MSC-EVs rescue disc degeneration by promoting the proliferation of degenerating nucleus pulposus cells and synthesizing the ECM synthesis through the miR-129-5p/SOX4/Wnt/β-catenin axis [66]. Consequently, MSC-EVs hold significant promise in modulating ECM synthesis to reverse osteoarthropathy.

### Regulating bone metabolism

Bone metabolism is intricately regulated by the balance between osteoblast-mediated bone formation and osteoclast-mediated bone resorption [67]. Imbalances, such as increased osteoclast activity and decreased osteoblast activity, can lead to delayed fracture healing or increased bone degeneration. The complex biological process of bone formation involves BM-MSC differentiating directly into osteoblasts through various biological factors. Notably, osteoblast-specific transcription factors, including runt-related transcription factor 2 (RUNX2) and osterix, play essential roles in osteoblast differentiation [68, 69]. Maintaining the balance of bone metabolism (osteoclast resorption and osteoblast remodeling) is vital for preventing the development of bone diseases. Increased osteolysis and bone resorption significantly impede bone growth into grafted tendons, affecting ECM synthesis and bone remodeling, ultimately leading to delayed early fracture healing [70]. Osteoclasts are the primary cell type responsible for the destruction and resorption of bone tissue in vivo, and inhibiting osteolysis while promoting osteogenesis and maintaining bone metabolism homeostasis are two central factors in promoting fracture healing and delaying joint degeneration [71].

Numerous preclinical and clinical studies have validated the potential of MSC-EVs in supporting bone healing by promoting osteogenic differentiation and inhibiting osteolysis. Feng et al. [72] proposed that miR-6924-5p, enriched in MSC-EVs, effectively inhibits tunnel osteolysis and enhances the biomechanical strength of tendon-bone healing by targeting two osteoclastic regulators, OCSTAMP and C-X-C motif chemokine ligand 12 (CXCL12). Simultaneously, MSC-EVs accelerated tendon-bone healing by delivering bone morphogenetic protein-2 (BMP-2), promoting the formation of bone and fibrocartilage tissues, as well as enhancing the stiffness and ultimate load strength of the tendon interface via the Smad/RUNX2 pathway [69]. Another intriguing study demonstrated that MSC-EVs loaded with recombinant C-Type lectin domain family 11, member A (CLEC11A) facilitate the transition of BM-MSC from lipogenic to osteogenic differentiation and inhibit osteoclast activity, ultimately alleviating osteoporosis [73]. Collectively, this evidence confirms that MSC-EVs can regulate bone metabolism to support fracture healing and alleviate osteoporosis. However, whether bone metabolism synergizes with biological behaviors such as angiogenesis and ECM degradation to improve the progression of related bone diseases is essential, as it may further explore the potential of MSC-EVs for clinical applications in orthopedic diseases [74].

### Regulating inflammatory response

Inflammatory responses are widely involved in the regulation of physiological and pathological processes in musculoskeletal disorders [75]. In the physiological state, inflammation is essential for tissue repair and regeneration, such as in fracture repair, as well as an indicator of bone and joint infection [75]. However, numerous studies have affirmed that inhibiting inflammation can alleviate chronic inflammation in degenerative musculoskeletal diseases such as OA and disc degeneration [76]. Notably, damage-associated molecular patterns (DAMP) activated by degenerating or stressed cells mediate persistent noninfectious inflammatory responses. The activation of the inflammatory response triggers the release of pro-inflammatory cytokines/chemokines from innate immune cells [77]. Mechanistically, elevated DAMP levels activate inflammatory vesicles, inducing caspase-1 activation and ultimately promoting the release of IL-1β and IL-18 [78]. Furthermore, active inflammatory diseases, including RA, may result from differences in the distribution of functional pro-inflammatory helper T cells (Th17) and anti-inflammatory regulatory T cells (Tregs) [79]. Therefore, strategies involving the elimination of DAMP-induced inflammatory responses and the modulation of immune responses hold potential for the clinical management of inflammatory diseases.

Studies have demonstrated that MSC can inhibit the inflammatory response by promoting anti-inflammatory processes during tissue repair, thereby creating an appropriate microenvironment for cartilage and musculoskeletal regeneration [80]. For instance, MSC induce the polarization of anti-inflammatory macrophages (M2- macrophages) and reduce levels of IL-1, IL-6, IL-8, IL-17, TNF-α, and IFN-γ in inflamed tissues [80–82]. Similar to their parental cells, MSC-EVs also exhibit promising potential in modulating inflammatory and immune responses. MSC-EVs ameliorate osteoporosis by inhibiting NOD-like receptor thermal protein domain associated protein 3 (NLRP3) inflammasome activation, as the negative regulation of NLRP3 inflammasome activation inhibits IL-1β and IL-18 secretion in osteoclasts, promoting recovery from bone loss [83, 84]. Additionally, macrophages have garnered attention for their potent immunomodulatory functions. MSC-EVs have been shown to reduce the inflammatory response of local tissues by polarizing macrophages from a pro-inflammatory M1 phenotype to an anti-inflammatory M2 phenotype, thereby promoting tendon-bone healing [57]. This may attributed to the M2 macrophage polarization, which decreases the expression levels of pro-inflammatory cytokines (IL-1β and IL-6) while stimulating the expression of anti-inflammatory cytokines (IL-10 and TGF-β) [85]. Given the immunomodulatory function of macrophage polarization, MSC-EVs may also suppress inflammation by inhibiting M1 macrophage polarization and promoting M2 macrophage infiltration [86]. Notably, specific signaling pathways may be associated with immune regulation in MSC-EVs. For instance, miR-23a-3p in BM-MSC-EVs supports tendon-bone healing by targeting and inhibiting IRF1 and NF-κB pathways in macrophages, promoting M2 macrophage polarization and suppressing inflammatory responses at the tendon-bone interface [87]. Furthermore, NF-κB is thought to be associated with GSDMD-mediated macrophage pyroptosis and inflammatory mediator release; however, the means by which MSC-EVs are involved in macrophage pyroptosis-based immune regulation are not yet known [88, 89]. Nevertheless, NF-κB may be a potential target for tendon-bone healing, and the detailed mechanisms by which MSC-EVs and NF-κB are involved in immune and inflammatory regulation must be further explored.

## Potential application of MSC-EVs as drug carriers

### Advantages of MSC-EVs as drug carriers

In comparison to synthetic biomaterials, EVs secreted by cells offer enhanced biocompatibility, lower immunogenicity, and greater target specificity [90]. The membranes of EVs are enriched with sphingolipids, signaling factors, and adhesion factors, facilitating their recognition and binding to target cells [91]. With a lipid molecular layer structure similar to cell membranes, EVs possess a robust ability to penetrate biological barriers (e.g., blood-brain barrier) and reach deeper tissues [92]. Additionally, the excellent tolerance of EVs allows them to evade immune phagocytosis, ensuring prolonged circulation [93]. These characteristics position EVs as effective drug carriers for delivering therapeutic agents to deep tissues.

In contrast to generic EVs, MSC-secreted EVs offer distinct advantages, rendering them exceptional drug carriers. MSC exhibit a heightened capacity to secrete EVs compared to other cells [94]. Concurrently, MSC-EVs demonstrate strong targeting abilities, and their low immunogenicity enables them to evade activating immune responses, resulting in a stronger circulating half-life [95]. Furthermore, MSC-EVs display effective lesion permeability and retention, allowing them to accumulate at the disease site [96, 97]. These unique attributes position MSC-EVs as highly valuable for clinical applications and ideal drug carriers for treating various diseases, including osteoarthropathies, neurodegeneration, and tumors.

### The application of MSC-EVs as drug carriers

Currently, various techniques are available for loading exogenous drugs into EVs and delivering them to specific lesions, including incubation, electroporation, ultrasonication, extrusion, and freeze-thaw cycles [98]. Among these methods, incubation is commonly used due to its operational simplicity, but its drug-loading efficiency limits further development [99]. Although electroporation exhibits higher drug loading efficiency than incubation, the electric charge may induce the denaturation of EV surface proteins and structural disruption [100]. Both sonication and extrusion methods result in higher drug loading efficiency than incubation. However, sonication affects EVs’ physicochemical properties and triggers protein aggregation, while inappropriate mechanical compression (extrusion) disrupts EVs’ membrane structural integrity [101, 102]. Freeze-thaw cycling has the potential for large-scale applications, but rapid and repeated cycles alter the physicochemical properties of surface proteins and remain less efficient than sonication for drug encapsulation [103, 104]. Overall, each drug loading method has its advantages and disadvantages, with electroporation being the most commonly used method for EVs, considering factors like convenience and cost [105].

Several reports confirm the therapeutic potential of different drug-loading methods for MSC-EVs in skeletal diseases. For instance, BM-MSC-EVs can be loaded with miRNA-542-3p by electroporation [106], and Li et al. [107] used incubation to load exogenous lncRNA CAHM into MSC-EVs, modulating macrophage polarization to ameliorate disc degeneration. Additionally, exogenous drugs were loaded onto MSC-EVs by extrusion and sonication to alleviate OA progression [108, 109]. These cases demonstrate the promising clinical applications of MSC-EVs as drug carriers.

## MSC-EVs targeted therapeutic potential

Notably, while the drug-loading efficiency of EVs can be improved in multiple ways, considering the prolonged retention of EVs in the blood circulation or tissues, improving the targeting of EVs would be beneficial to increase the efficiency of drug therapy. Current research focuses on improving EV targeting through engineering modifications.

### Natural bone targeting capacity of MSC-EVs

Natural EVs released from cells inherently possess the ability to deliver their contents to host cells. Membrane surface proteins of EVs, such as tetraspanning proteins (CD9, CD63, and CD81), latex adhesion proteins (LA), Lamp-2b, and heat shock proteins (HSP), contribute to the targeting of EVs to host cells and mediate their entry into the recipient cells [110, 111]. Theoretically, EVs can be specifically internalized in a cell type-specific manner, depending on the identification of ligand proteins on the EV surface by the cell or tissue [112]. For instance, the interaction of CXC chemokine receptors (CXCR4) with stromal cell-derived factor 1 (SDF-1) can facilitate the targeted metastasis of MSC-EVs to osteosarcoma sites [113]. Furthermore, EVs secreted by different cells may contain signaling molecules that act as ligands for other cells, and these receptor-ligand interactions facilitate the enrichment of EVs in specific cells or tissues [114]. Zhang et al. [115] demonstrated that EVs derived from BM-MSC could target miR-206 for translocation into osteosarcoma tissues and regulate tumor growth. Induced by chemokines, MSC-EVs may accumulate in specific tissues through vascular leakage, revealing the homing effect of MSC [116]. The promising tissue-targeting properties of MSC-EVs hold much promise for the study and treatment of bone diseases, although more research is still worth exploring.

### Engineering modifications of MSC-EVs to enhance bone targeting capability

Considering the inefficiency of the natural targeting of MSC-EVs, enhancing the bone targeting of MSC-EVs through engineering modifications is a potential option to improve the efficiency of bone disease treatment.

#### Direct modification of MSC-EVs

Click chemistry, membrane postinsertion, hydrophobic insertion, and receptor-ligand binding are typical approaches for direct modification of bone-targeted MSC-EVs.

Click chemistry involves coupling ligands to the outer surface of EVs, which is achieved by direct modification of EVs [117]. For example, EVs binding to alendronate can generate Ale-EVs through “click chemistry,” facilitating EV targeting to the bone via alendronate/hydroxyapatite binding and ultimately alleviating osteoporosis [118]. Copper-catalyzed azide-alkyne cycloaddition reactions (click chemistry) were utilized by Smyth et al. for bioconjugation of specific molecules to the surface of EVs, confirming its potential for improving bone-targeting ability [119].

Membrane postinsertion operation, due to its simplicity and flexibility, is considered an attractive EV modification strategy. This approach achieves covalent attachment of the target ligand to polyethylene glycol-lipid micelles, which are then co-incubated with selected liposomes before transferring to the liposome bilayer [120]. Yan et al. [121] demonstrated that this modification, involving folic acid (FA)-polyethylene glycol-cholesterol compound, enhances bone-targeting of EVs encapsulating dexamethasone sodium phosphate, contributing to RA amelioration. Incorporating folic acid onto EVs via membrane postinsertion strategy improves bone targeting and reduces synovial inflammation, showing good biocompatibility and extended circulating half-life [122–124].

Hydrophobic interactions have been employed to modify the surface of EVs through the insertion of hydrophobic diacyl lipids, facilitating the modification of EVs with bone-targeted peptides [125]. In this method, bone-targeting peptides are incubated with EVs, and hydrophobic interactions are harnessed to craft EVs capable of bone-targeting delivery. This approach involves modifying EVs through hydrophobic interactions and loading Shn3-expressing siRNAs into MSC-EVs by demonstrating the inherent bone-targeting properties of MSC-EVs, thereby further enhancing the therapeutic efficacy of osteoporosis treatment, as well as promoting osteoclast differentiation and facilitating angiogenesis [125, 126].

Currently, researchers employ receptor-ligand interactions to affix natural receptors onto the surface of EVs, creating targeted ligands [127]. Leveraging the selectivity of aptamers, various techniques are employed to modify the EV surface, enhancing bone targeting. Luo et al. [128] demonstrated that coupling of bone marrow stromal cell-derived EVs with BM-MSC-specific aptamers enables the targeted delivery of EVs to BM-MSC within the bone marrow. This approach avoids immune clearance and metabolism, thus offering potential options for treating osteoporosis and fractures. The glycoproteins present on EV membranes make glycosylphosphatidylinositol (GPI) an anchoring structure for functional ligands on the surface of EVs. The aptamer modification using GPI protects EV surface proteins from proteolytic degradation by proteases, thereby improving the targeting ability of EVs [129]. Furthermore, co-incubation of aldehyde-modified aptamers with BM-MSC-EVs facilitates the synthesis of aptamer-functionalized EVs, contributing to bone targeting and promoting bone repair and regeneration [128, 130].

#### Indirect modification of MSC-EVs

Recognizing the analogous properties of EVs to parental cells, a viable strategy for obtaining targeted EVs involves the genetic engineering modification of parental cells [131]. The modification of parental cells that produce EVs, such as directing signaling peptides to the surface of EVs, is achieved using plasmid vectors encoding target ligands fused to transmembrane proteins [110]. Lamp-2b, serving as a surface protein with a signal peptide for EVs, is often fused to targeting proteins to present the protein as a targeting site, ligand, or receptor on the EV surface. For instance, Liang et al. stably transfected dendritic cells with the CAP-Green fluorescent protein (GFP)-Lamp2b plasmid, obtaining EVs specifically targeted to chondrocytes. These EVs delivered miRNA-140 to the deep cartilaginous region by effectively targeting chondrocytes [132]. Similarly, chondrocyte-targeted EVs were engineered by genetically fusing a chondrocyte affinity peptide to the N-terminal end of the EV surface protein Lamp2b, demonstrating favorable osteoarticular targeting [133]. EVs obtained through genetic engineering of parental cells not only retained their bone-targeting properties but also exhibited increased stability. However, the degradation of peptides by endoplasmic proteases within cells poses a significant challenge to the yield of targeted peptide-functional EVs.

Indeed, specific metabolic reprogramming can enhance the acquisition of targeted EVs. For instance, metabolic glycoengineering (MGE) facilitates biorthogonal copper-free click chemical modification on the parental cell surface [134]. Dong et al. [135] utilized MGE by culturing parental cells with tetraacetylated N-azidoacetyl-D-mannosamine to generate unnatural azide moieties onto their membrane surfaces. Subsequently, dibenzocyclooctyne-coupled dextran sulfate (DBCO-DS) was attached via click chemistry to azide-containing parental cells, resulting in the creation of macrophage-targeted EVs. This innovative targeting modification holds promise for advancing orthopedic disease treatment by allowing the incorporation of targeting molecules into EVs, thereby achieving bon-specific targeting while maintaining structural stability.

### MSC-EVs combined with biomaterials to improve bone targeting capabilities

MSC-EVs can be directly employed in bone disease treatment, typically through the injection of an aqueous solution containing MSC-EVs into the circulation or tissue to enhance tissue repair. However, the direct injection of free MSC-EVs in aqueous solutions poses challenges, as they are difficult to retain in the target region for extended periods and may be promptly removed, hindering the full utilization of their biological functions [136]. Consequently, direct injection of MSC-EVs in an aqueous solution may not be an optimal strategy. Currently, numerous studies have reported cases where MSC-EVs are combined with biomaterials to enhance clinical therapeutic efficacy. This approach aims to confer MSC-EVs with the capability of controlled drug release at the disease site in a dose- and time-dependent manner [136, 137]. The ideal biomaterials for combination with MSC-EVs should possess the following properties: I. The in vivo degradation rate of the material should not be excessively fast, ensuring the delivery of MSC-EVs to the target tissues and allowing control over the release rate of the loaded drug [138]. II. After the release of the MSC-EVs loaded drug, the biomaterial should degrade within the tissue, and the degradation rate should align with the regeneration rate. III. The biomaterials should be targeted and can be co-targeted with MSC-EVs for efficient delivery to the disease site [139].

Hydrogels, owing to their excellent biocompatibility, biodegradability, and advantageous properties for cell infiltration and adhesion, have been increasingly utilized in scientific research and clinical application [140]. Studies indicate that biomolecules bound to hydrogels maintain their structure and function for an extended period, attributed to the hydrogel’s ability to remodel the microenvironment, facilitating the storage and uptake of MSC-EVs while enabling targeted delivery to bone tissue [141]. For example, hydrogels loaded with MSC-EVs exhibit the capacity to target bone tissue, promoting the repair of bone tissue defects and mitigating disc degeneration [142, 143]. Mechanistically, the synergistic combination of hydrogels and MSC-EVs may offer enhanced efficacy for alleviating bone diseases by modulating angiogenesis, ameliorating inflammation, and remodeling the immune environment [140, 144]. Additionally, other biomaterials, including degradable implants, tissue-derived materials, and growth factors, may offer potential options to improve the targeting and therapeutic efficiency of MSC-EVs.

## Therapeutic applications of MSC-EVs in bone-related diseases

**Fig. 2:**
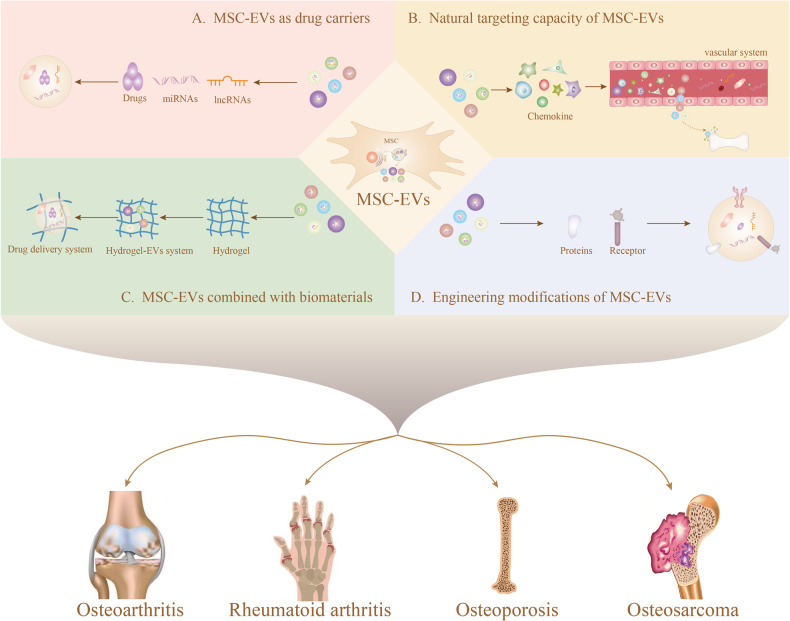
The clinical potential of MSC-EVs is multifaceted. They serve as drug carriers, have natural targeting capacity-engineered modifications, and exhibit synergy when combined with biomaterials in bone diseases.

Considering MSC-EVs’ ability to target specific cells or tissues and minimize the risk of toxicity to non-target tissues, they have been designed as a novel drug carrier for direct drug delivery to bone tissues, addressing various bone-related diseases, including OA, osteoporosis, RA, osteosarcoma (Fig. 2) (Table 1).

**Table 1:** Therapeutic potential of MSC-EVs for bone-related diseases.

Cell Source	Content	Engineered modification	Bone disease	Main Finding	Reference
BM-MSCs	miR15a-5p miR16-5p	Parental cell modification	Bone injury	BM-MSCs-derived EVs are engineered to enhance bone targeting for enhanced bone repair and bone regeneration	[167]
MSC	miR-21-5p	None	Anterior cruciate ligament injury	MSC-EVs promote tendon-bone healing through miR-21-5p/SMAD7 signaling	[168]
BM-MSCs	miR-6924-5p	Expression of PDGFRα (+) on EVs membranes	Tendon-bone injury	BM-MSC-EVs loaded with miR-6924-5p as a potential therapy for osteolysis during tendon healing and improved prognosis	[72]
MSC	miR-410	None	Intervertebral disc degeneration	MSC-EVs inhibit cellular pyroptosis via miR-410 to alleviate intervertebral disc degeneration	[169]
MSC	miR-4450	None	Intervertebral disc degeneration	MSC-EVs upregulate ZNF121 via miR-4450 to alleviate disc degeneration	[170]
BM-MSCs	Not given	Binding to hydrogels	Osteonecrosis	Engineered MSC-EVs support bone repair through osteogenesis and angiogenesis	[171]
MSC	pBMP2	Parental cell modification	Inadequate bone healing	Engineered MSC-EVs promote bone regeneration and osteogenic differentiation to accelerate bone healing	[172]
MSC	netrin-1	Loading netrin-1	Spinal cord injury	Engineered MSC-EVs enriched with netrin-1 modRNA promote axonal growth to restore spinal cord injury	[173]
BM-MSCs	antagomir-188	Fusion with liposomes	Osteoporosis	Engineered MSC-EVs target bone tissue via CXCR4 to regulate bone metabolism and alleviate osteoporosis	[150]
MSC	miR-205-5p	Fusion with hydrogel	Bone injury	MSC-EVs promote cartilage regeneration via the miR-205-5p/PTEN/AKT pathway after binding to hydrogels	[39]
MSC	Shn3	Loading siRNA	Osteoporosis	Bone-targeted engineered MSC-EVs treat osteoporosis by delivering siRNAs	[125]
MSC	Doxorubicin	None	Osteosarcoma	MSC-EVs inhibit osteosarcoma proliferation by delivering doxorubicin	[174]
BM- MSCs	Not given	Fusion with hydrogel	Spinal cord injury	MSC-EVs combined with hydrogels for the treatment of spinal cord injuries	[175]
MSC	Not given	None	Osteoarthritis	BM-MSC-EVs protect against cartilage damage and alleviate osteoarthritis	[163]
MSC	miR-129-5p	None	Osteoarthritis	MSC-EVs target HMGB1 via miR-129-5p to alleviate IL-1β-induced osteoarthritis	[176]

### Osteoarthritis

Osteoarthritis (OA), a prevalent joint disease, is characterized by articular cartilage damage involving degeneration, fibrosis, fractures, defects, and overall joint surface impairment [145]. MSC-EVs emerge as promising drug carriers due to their capacity to deliver contents precisely to chondrocytes. For instance, MSC-EVs ameliorate OA by targeting histone deacetylase 3 and STAT1/NF-κB p65, delivering miR-326 to chondrocytes and cartilage, and inhibiting cell death within cartilage [146]. However, traversing the dense ECM of cartilage for molecules like miRNA remains challenging. Thus, the use of engineered EVs for delivery becomes a potential option. Liang et al. [133] engineered EVs by attaching chondrocyte affinity peptides to the N-terminal end of the EVs surface protein Lamp2b. Subsequently, they employed liposome membrane fusion to form hybridized chondrocyte-targeted EVs, encapsulating the CRISPR/Cas9 plasmid. The results demonstrated that such engineered EVs effectively targeted chondrocytes within cartilage injury, mitigating the degradation of ECM proteins and ultimately alleviating OA.

### Osteoporosis

Osteoporosis, a systemic metabolic bone disease, primarily results from bone mass loss, destruction of bone tissue microstructure, and increased bone fragility, leading to fractures in patients [147]. The root cause of osteoporosis lies in the imbalance between osteoblasts and osteoclasts, disrupting bone metabolism and a shift in bone marrow cell lineage from osteoblasts to adipocytes. Current osteoporosis drugs, such as bisphosphonates, estrogens, and calcitonin, lack bone tissue targeting, resulting in poor therapeutic efficacy and associated side effects like nephrotoxicity and jaw necrosis [148, 149]. Given the excellent bone tissue targeting properties of MSC-EVs, they have been applied as drug carriers for osteoporosis treatment. For example, MSC-EVs-loaded LncRNA MALAT1 can target bone tissue by mediating the miRNA-34c/SATB2 axis, effectively alleviating osteoporosis [30]. Engineering modifications can further enhance the bone-targeting ability of MSC-EVs, thereby improving the therapeutic efficiency of osteoporosis drugs. The coupling of BM-MSC-EVs with BM-MSC-specific aptamers enhances the bone targeting of EVs, facilitating targeted drug delivery to bone tissue, promoting bone regeneration, and ameliorating osteoporosis [128]. Similarly, utilizing CXCR4-based EVs in combination with nanoparticles, expressing CXCR4 in the nature of EVs enables bone-targeted drug delivery, which could accumulate and release drugs in the bone marrow to reverse aging-related bone loss [150]. In summary, drug carriers based on MSC-EVs present a promising strategy against osteoporosis.

### Rheumatoid arthritis

Rheumatoid arthritis (RA) is a chronic autoimmune disease with systemic involvement, characterized by joint inflammation, destruction of bone and cartilage, and loss of function [151]. Despite the use of NSAIDs, disease-modifying anti-rheumatic drugs (DMARDs), and biologics (e.g., TNF-α antagonists, IL-1 receptors and IL-6 receptors) in RA treatment, factors such as lack of specificity and low bioavailability continue to emphasize the need for innovating therapeutic approaches [152, 153]. Fortunately, MSC-EVs play a role in the amelioration of RA and can suppress inflammation through factors such as IL-1. This mechanism might be related to MSC-EVs’ ability to regulate the immune microenvironment by influencing the numbers of T cell and B cell subsets [154, 155]. Additionally, effective modulation of pro-inflammatory M1 and anti-inflammatory M2 macrophages, crucial in the inflammatory and autoimmune responses of RA, can mitigate the disease progression [156]. In a study, surface modification of macrophage-derived EVs with the anti-inflammatory cytokine interleukin-10, coupled with the encapsulation of the chemotherapeutic agent betamethasone sodium phosphate in EVs, facilitated targeted delivery to bone tissue, promoting M2 macrophage polarization and improving rheumatoid joint progression [157]. However, few studies have been reported on the use of engineered modified MSC-EVs for RA therapy. Considering the immunomodulatory capabilities of both MSC-EVs and macrophages, drug delivery to bone tissue through engineering modifications to MSC-EVs may present a potential therapeutic modality for RA [73, 158]. While more experiments are necessary to validate this speculation, natural MSC-EVs, as drug carriers, exhibited promising therapeutic effects in RA treatment [159].

### Osteosarcoma

Osteosarcoma is a malignant tumor arising from mesenchymal cells, typically occurring in adolescents. Paradoxically, studies have revealed that MSC-EVs are involved in the dual regulation of osteosarcoma progression. On the one hand, they promote the proliferation and metastasis of osteosarcoma while simultaneously inhibiting its progression. For instance, lncRNA XIST in MSC-EVs can promote osteosarcoma growth and metastasis through the miR-655/ACLY signaling [41]. Conversely, miRNA-150 carried by MSC-EVs inhibits the proliferation and migration of osteosarcoma cells by regulating IGF2BP1 [160]. The contradiction arises from the diverse contents carried out by MSC-EVs. Nevertheless, it is undeniable that MSC-EVs can serve as effective drug carriers for targeted osteosarcoma treatment. For example, loading doxorubicin into MSC-EVs facilitates osteosarcoma therapy, with doxorubicin-loaded MSC-EVs targeting osteosarcoma therapy via the SDF1-CXCR4 axis [90, 113]. While there is a shortage of studies on the use of engineered MSC-EVs specifically for osteosarcoma treatment, insights from related cases can offer valuable perspectives. Huang et al. [161] demonstrated the preparation of c(RGDyK)-modified and MEG3-loaded EVs to enhance their targeting of osteosarcoma, effectively inhibiting osteosarcoma progression. This suggests the potential application of engineered MSC-EVs in advancing targeted therapies for osteosarcoma.

Importantly, the efficacy of MSC-EVs for the treatment of bone disease needs to be confirmed by more clinical studies. Currently, OA-based clinical studies have focused on MSC, and clinical studies have demonstrated that MSC can alleviate the symptoms of OA and promote cartilage regeneration [162, 163]. In addition, clinical studies have been used to confirm the efficacy of MSC-EVs in the treatment of OA, and unfortunately these two clinical studies have not yet been completed (NCT05520125, NCT04998058) [164]. Similar to OA, research on degenerative disc disease has focused on MSC and clinical studies have demonstrated that MSC can relieve back pain and slow the progression of degenerative disc disease (NCT04499105, NCT04759105) [165]. In addition, a Phase I clinical study of MSCEVs for the treatment of degenerative disc disease is ongoing and has not yet reached definitive conclusions (NCT04849429). For osteosarcoma, MSC-EVs have mainly been used as biomarkers for tumor diagnosis, and a clinical study at Ruijin Hospital in China confirmed the promising diagnostic potential of MSC-EVs in osteosarcoma (NCT05101655, NCT03108677) [166]. Considering that MSC-EVs have similar properties to MSC, MSC-EVs have a promising future for the treatment of bone diseases, but more research is needed to promote the clinical study and application of MSC-EVs for the treatment of bone diseases.

## Conclusion and future perspectives

MSC-EVs are considered an effective tool for promoting tissue repair and regeneration. In recent years, extensive research has delved into the therapeutic potential of MSC-EVs in treating bone-related disorders. Their mechanisms and clinical applications have been widely explored. Functioning as bio messengers loaded with genetic material, MSC-EVs play a crucial role in regulating the progression of bone-related diseases. This involves actions such as promoting angiogenesis and maintaining bone metabolic homeostasis through miRNAs and lncRNAs, thereby alleviating conditions like OA, RA, and osteoporosis. Capitalizing on their exceptional properties, including high biocompatibility, low immunogenicity, easy penetration of biological barriers, and prolonged circulating half-life, MSC-EVs are acknowledged as promising vehicles for treating bone-related diseases. They can alleviate the disease symptoms by carrying various loads, such as NSAIDs, chemotherapeutic agents, and miRNAs. However, recognizing the inherent limitation of natural MSC-EVs in low bone targeting, their efficacy can be significantly enhanced through diverse engineering modification strategies. These modifications aim to improve the precision of drug delivery, increase loading efficiency, enable controlled release, and improve bone targeting. MSC-EVs exhibit the potential to revolutionize the treatment of bone-related diseases, thus a novel and promising therapeutic strategy.

However, the clinical application of MSC-EVs faces several challenges. First, there is a pressing need for effective procedures to purify clinical-grade MSC-EVs from samples, necessitating the development of robust purification guidelines. Second, the evaluation and analysis of heterogeneity within subpopulations of MSC-EVs pose challenges, given that individual MSC-EVs loaded with different biomolecules can significantly impact tissue biological functions. Thirdly, the administration route, optimal dosage, efficiency, and cost-effectiveness of MSC-EVs as drug carriers require thorough evaluation. Additionally, feasible strategies must be identified for engineering modifications on a large scale to enhance the therapeutic applications of MSC-EVs.

Furthermore, more extensive clinical research is essential to confirm the clinical utility and safety of bone-targeted MSC-EVs, especially when combined with biomaterials and promising AI strategies. The paradoxical role of MSC-EVs in regulating osteosarcoma progression, simultaneously promoting tumor proliferation and metastasis while inhibiting tumor progression, adds complexity. Consequently, careful consideration is necessary to avoid potential tumorigenic effects when utilizing MSC-EVs as drug carriers for osteosarcoma treatment. Despite these challenges, the potential of bone-targeted MSC-EVs as a next-generation therapeutic platform for bone-related diseases remains promising.
